# Paving the Way for Motor Imagery-Based Tele-Rehabilitation through a Fully Wearable BCI System †

**DOI:** 10.3390/s23135836

**Published:** 2023-06-23

**Authors:** Pasquale Arpaia, Damien Coyle, Antonio Esposito, Angela Natalizio, Marco Parvis, Marisa Pesola, Ersilia Vallefuoco

**Affiliations:** 1 Department of Electrical Engineering and Information Technology (DIETI), Università Degli Studi di Napoli Federico II, 80125 Naples, Italy; marisa.pesola@unina.it; 2 Augmented Reality for Health Monitoring Laboratory (ARHeMLab), Università Degli Studi di Napoli Federico II, 80125 Naples, Italy; antonio.esposito9@unina.it (A.E.); angela.natalizio@polito.it (A.N.); ersilia.vallefuoco@gmail.com (E.V.); 3 Centro Interdipartimentale di Ricerca in Management Sanitario e Innovazione in Sanità (CIRMIS), Università Degli Studi di Napoli Federico II, 80125 Naples, Italy; 4 Institute for the Augmented Human, University of Bath, Bath BA2 7AY, UK; dh.coyle@ulster.ac.uk; 5 Intelligent Systems Research Centre, University of Ulster, Derry BT48 7JL, UK; 6 Department of Electronics and Telecommunications (DET), Politecnico di Torino, 10129 Turin, Italy; marco.parvis@polito.it; 7 Department of Psychology and Cognitive Science, University of Trento, 38122 Rovereto, Italy

**Keywords:** electroencephalographic sensor, dry sensors, motor imagery, brain–computer interface, neurofeedback, tele-rehabilitation

## Abstract

The present study introduces a brain–computer interface designed and prototyped to be wearable and usable in daily life. Eight dry electroencephalographic sensors were adopted to acquire the brain activity associated with motor imagery. Multimodal feedback in extended reality was exploited to improve the online detection of neurological phenomena. Twenty-seven healthy subjects used the proposed system in five sessions to investigate the effects of feedback on motor imagery. The sample was divided into two equal-sized groups: a “neurofeedback” group, which performed motor imagery while receiving feedback, and a “control” group, which performed motor imagery with no feedback. Questionnaires were administered to participants aiming to investigate the usability of the proposed system and an individual’s ability to imagine movements. The highest mean classification accuracy across the subjects of the control group was about 62% with 3% associated type A uncertainty, and it was 69% with 3% uncertainty for the neurofeedback group. Moreover, the results in some cases were significantly higher for the neurofeedback group. The perceived usability by all participants was high. Overall, the study aimed at highlighting the advantages and the pitfalls of using a wearable brain–computer interface with dry sensors. Notably, this technology can be adopted for safe and economically viable tele-rehabilitation.

## 1. Introduction

Tele-rehabilitation has long been considered a promising way of providing rehabilitative therapies “at distance” [1,2,3]. Digital sensing and artificial intelligence solutions enable patient-centered treatment by continuously monitoring and evaluating patient performances [4,5]. Over the past few years, the COVID-19 pandemic has accelerated this transition to a new era known as health 5.0 [6,7]. In this context, extended reality helped to provide an alternative therapy at a distance for a wide range of people. Notably, different solutions were proposed for older adults with neurodegenerative diseases [8,9,10].

Brain–computer interfaces (BCIs) based on the motor imagery paradigm have been extensively studied for human patients with a variety of neuromuscular disorders in order to facilitate the recovery of neural functions. Their effectiveness has been confirmed, especially for stroke patients [11,12,13]. The combination of BCIs and extended reality can provide patients with neurofeedback on their mental tasks [14]. In particular, sensory feedback helps them in the self-regulation of brain rhythms and promotes neural plasticity.

The literature has shown that neurofeedback improves classification for motor imagery and that sensorimotor cortical activation is significantly enhanced. This was quantified not only in terms of classification accuracy, with an improvement of about 10% to 20% [15,16], but also as event-related spectral perturbation and functional connectivity [15].

To be involved in tele-rehabilitation, a system including a BCI and extended reality must be non-invasive, wearable, portable, comfortable, and generally ready for getting out of controlled lab environments [17,18]. Moreover, wireless features are desirable in disclosing new applications with brain-type communication services [19]. These requirements are often fulfilled by exploiting electroencephalography (EEG) to acquire brain signals [20]. EEG systems for “out-of-lab” acquisitions are increasingly being developed [21]. These are mainly wireless devices with a reduced number of sensors that allow freedom of movement and improve usability [22,23]. Moreover, instead of the standard wet sensors, dry sensing could be used to increase user comfort while attempting to keep a high metrological performance [24,25,26].

Previous studies already proposed EEG devices relying on dry sensors. They relied either on ad hoc instrumentation [27,28,29] or evaluated consumer-grade instrumentation [30,31] involving dry electrodes. For instance, in [32], classification was attempted in different dry sensing setups (from 8 to 32 sensors) and with different signal processing approaches. A wireless high-density EEG medical grade system was used and a drop in performance was observed when eight channels were used. However, neurofeedback was not investigated in trying to enhance motor imagery detection. Recently, the feasibility of a wearable BCI for neurorehabilitation at home was proposed in [33]. Healthy participants received remote instructions on the use of an EEG device with 16 dry sensors. Visual feedback consisted of a bar fluctuating vertically up or down from the midline. Half of the participants succeeded in controlling the BCI during six sessions.

It is worth noting that a previously published work [16] already investigated a similar motor-imagery-based BCI with wet sensors. Moreover, unimodal feedbacks (visual and haptic) were investigated along with multimodal visual–haptic feedback. The results highlighted the role of neurofeedback in improving the performance, and participants generally preferred visual and visual–haptic feedback modalities. Nonetheless, the experiments had to be extended to a greater number of participants.

The aim of the present study was to prototype and validate a user-friendly BCI that could then address tele-rehabilitation. This was performed by emphasizing wearability, comfort, engagement, and ease of use. An upgraded version of a previously proposed system [16] was designed and developed incorporating a ready-to-use Class IIA EEG device with eight dry sensors, certified according to the Medical Device Regulation. The effectiveness of visual–haptic neurofeedback in discriminating between left hand and right hand motor imagery was also investigated over 5 experimental sessions for each of the 27 enrolled subjects. Notably, this multimodal feedback was chosen in accordance with the subjects’ preference proven by the previous study [16]. To this aim, the subjects were divided into a control group and a neurofeedback group. Preliminary results were presented in [34] but extended here by considering a large subject cohort and the results of questionnaires administered to evaluate usability. The remainder of the paper is organized as follows: Section 2 presents an overview of the proposed system, with a focus on the experimental protocol and outcome measures; Section 3 shows the system performance in experiments; and Section 4 concludes the manuscript by discussing the results.

## 2. Materials and Methods

This section discusses the design, implementation, and validation methods for a wearable BCI relying on motor imagery, EEG with dry sensors, and online neurofeedback. An overview of the system is given together with the adopted hardware. Then, EEG processing is focused in association with the experimental protocol. Questionnaires will also be introduced. They were adopted to assess the usability of the system and imaginative abilities of its users. Finally, the tests considered within the statistical analysis are recalled.

### 2.1. System Overview

**Figure 1:**
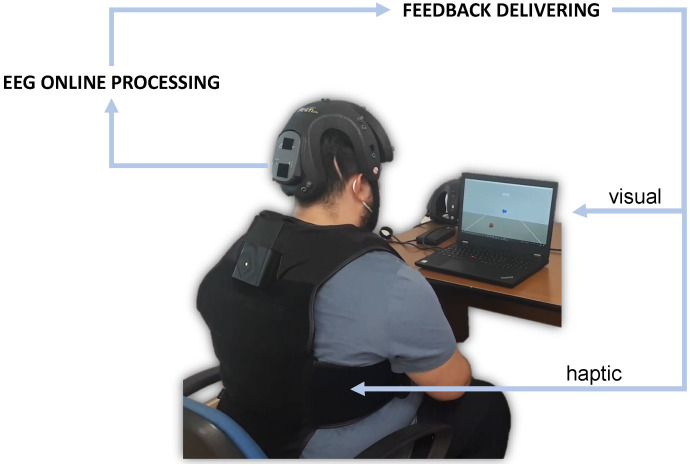
A subject using the proposed BCI system with neurofeedback in extended reality. The system involves EEG acquisition with the Helmate device, online processing, and actuators for visual–haptic feedback delivery.

The present study proposes a new system integrating a BCI with neurofeedback in extended reality, where a virtual reality environment was set to provide both visual and haptic virtual sensations (Figure 1). This could be addressed both to daily-life applications for tele-operating a device [19,35] and to tele-rehabilitation purposes.

**Figure 2:**
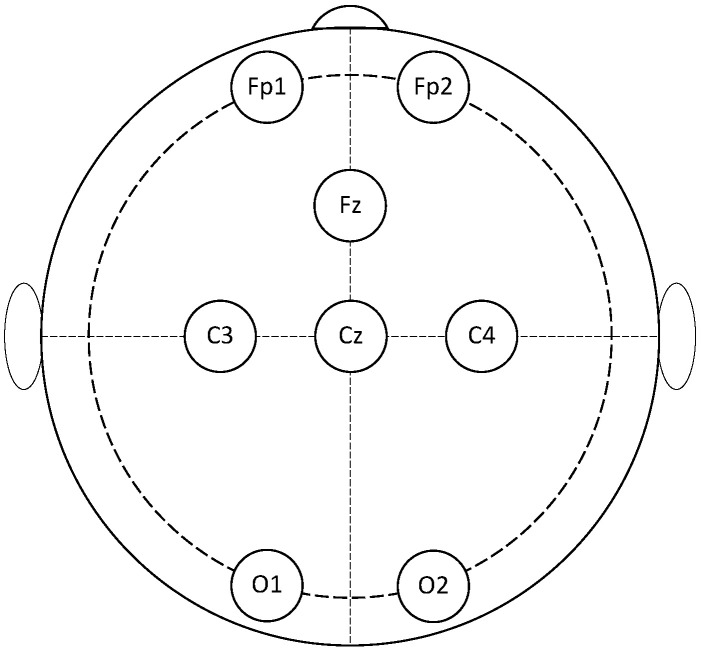
Position on the scalp of the sensors adopted in this study. Locations are identified by the 10–20 standard system for EEG.

In the system, brain signals were acquired by using the Helmate EEG device by ab medica (https://www.abmedica.it/, accessed on 12 Febraury 2023). This is a Class IIA device certified according to the Medical Device Regulation (EU) 2017/745. It has eight measuring channels plus one reference channel and one bias channel. Ten dry sensors with different shapes can be chosen according to the zone of the scalp to reach. Moreover, as a multipurpose device, different configurations for the channels’ location could be exploited. In this study, the eight measuring channels were located at FP1, FP2, Fz, Cz, C3, C4, O1, and O2 according to one of the default configurations, while the reference and bias sensors were placed in the frontal region at AFz and FPz, respectively (Figure 2). Such a configuration guarantees the optimal mechanical stability of the helmet during measurements. Moreover, it makes the system open to future upgrades by allowing, for instance, the integration of a module for monitoring users’ engagement.

Data were collected at a sampling rate of 512 Sa/s and transmitted via Bluetooth to a custom Simulink model for EEG processing. In Simulink, features from the EEG signal were extracted by means of the filter bank common spatial pattern (FBCSP) [36] and classified by means of the naive Bayesian Parzen window (NBPW). The latter returns two outputs: the class to which the multichannel EEG signal is assigned (right or left) and the probability associated with that class.

**Figure 3:**
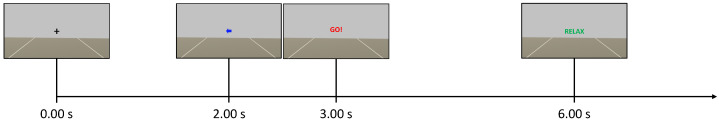
Timing of a single trial of the experimental sessions for the control group. The same timing was also used for the neurofeedback group only during the first phase of an experimental session. Notably, there was an overlap of 0.25 s between the cue and the word “GO!”.

The classification outputs were used to drive multimodal feedback through a custom Unity application. The neurofeedback consisted of a combination of visual and haptic feedback associated with the mind control of a virtual object and coherent tactile sensation. For visual feedback, a virtual ball was shown on a display (Figure 3). This could roll to the left or to the right of the virtual environment according to the EEG classification. In detail, while the assigned class determined the direction, the related score determined its velocity. The TactSuit X40 from bHaptics Inc was used for the haptic feedback. This is a wearable and portable vest equipped with 40 individually controllable vibrotactile motors. The vibration was again modulated by classification outputs. More specifically, the pattern could move from the center of the torso (front side) to the right or to the left in accordance with the assigned class. Meanwhile, the related score determined the vibration intensity. It is worth noting that only the bottom motors were used to minimize vibration artifacts on the EEG signals.

### 2.2. Experimental Protocol

The described BCI was exploited within a cue-based (synchronous) paradigm. This implied that the user had to imagine a movement or be relaxed in accordance with given indications (the cues). The indications were delivered visually by means of the Unity3D platform. Two motor imagery tasks were possible, namely imagining the movement of the left hand or imagining the movement of the right hand. In case of neurofeedback, multimodal feedback was delivered to the user in response to the ongoing mental task. It should be noted that this was not simply intended for training the user (i.e., neurofeedback training) but as a part of the BCI online operation. Indeed, the actual role of this neurofeedback was to enhance the users’ experience by providing some information on the ongoing brain activity. On the other side, the classifier adopted for the online processing had to be identified. This was performed by exploiting signals acquired during pure motor imagery (no feedback).

In the experimental protocol, subjects were divided into two groups and involved in five one-hour experimental sessions over five weeks. The subjects assigned to a control group never received feedback. For the subjects of the neurofeedback group, pure motor imagery had to be recorded at the beginning of each session, and then neurofeedback was provided thanks to an EEG classifier trained on these preliminary data. The protocol for the two groups is described in detail in the following.

#### 2.2.1. Control Group

The Unity application dictated the timing within the experimental session. A total of 6 runs were recorded, and each run consisted of 30 trials. Each trial consisted of a fixation cross visualized from 0.00 s to 2.00 s, a cue (left or right arrow) visualized from 2.00 s to 3.25 s, the word “GO!” visualized from 3.00 s to 6.00 s, and the word “RELAX” visualized for a random time window of 1.00 s to 2.00 s (Figure 3). Notably, words were displayed to guide the user through the experiment in the absence of any feedback on the screen. The sequence of left and right cues and the duration of the final “RELAX” were randomized across trials to avoid biases. The EEG was acquired as a continuous stream during each run but never processed online and thus the control group did not receive any feedback. The runs were separated by short breaks, with a longer time break between the first three runs (phase 1) and the last three runs (phase 2) of a session.

#### 2.2.2. Neurofeedback Group

**Figure 4:**
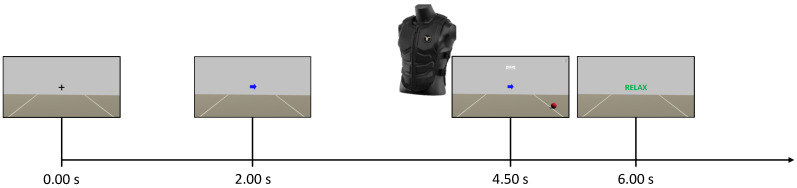
Timing of a single trial of the experimental sessions for phase 2 of the neurofeedback group. The same timing of the control group was used for phase 1.

The first three runs of each session (phase 1) were carried out as they were for the control group. However, during the time break between the phases, the EEG data from phase 1 were used to train the online classifier. This classifier was trained from scratch for each subject and for each session. Subsequently, the participants of this group performed three further runs (phase 2), during which they received online multimodal feedback in response to motor imagery. The goal of the participants in the neurofeedback group was to move the visual feedback ball over the white lines of the game environment and to maximally activate the motors of the vest on the back of the respective side (i.e., left or right). In this experimental phase, words were no longer appearing but the user was fully guided by the arrows and the virtual ball (Figure 4). In this case, the timing was slightly changed because participants were asked to start imagining from the appearance of the cue at t= 2.00 s. Then, they received the feedback from 4.50 s to 6.00 s (Figure 4). The instant t= 4.50 s depended on the fact that the system actually started to classify at t= 2.50 s, and the time window for online processing was 2.00 s wide. Finally, the feedback could only move if the label obtained from the online classifier matched the assigned task (positive bias). Otherwise, no feedback was provided and the virtual ball was dragged towards the center of the screen while the intensity of the vibration was interrupted. Further details on that are discussed in the next subsection.

### 2.3. EEG Processing

The FBCSP with the NBPW classifier was used not only for online processing but also for the offline processing of EEG data. This is a well-known approach in the literature of motor imagery BCIs [36] and it is still considered one of the most successful ones for binary classification [37]. Its main blocks involve the following:

1. Time domain filtering by means of a filter bank (FB) with 17 overlapped bandpass Type II Chebyshev filters with order 10 from 4 Hz to 40 Hz;
2. Features extraction using a spatial domain filtering by means of the common spatial patterns (CSPs) algorithm [35];
3. Feature selection based on the class-related information content of the features using the mutual-information-based best individual features selector;
4. Feature classification exploiting the Bayesian (NBPW) classifier.

Further details on the processing pipeline can be found in [16,34,36]. With reference to the neurofeedback group, after acquiring the EEG in a first half-session, data processing was needed to train the online classification algorithm. Specifically for online processing, the FBCSP-based approach was adapted so that the EEG stream was processed with a sliding window covering the motor imagery period.

Through exploiting the results of previous studies [16,34], the time-width for the sliding windows was fixed at 2.00 s, and this was used to span the interval from 0.00 s to 7.00 s with a 0.25 s shift. A five-folds cross validation with five repetitions was used to identify the best portion of the EEG trials for training the algorithm. This best 2.00 s wide window was selected as the one associated with the highest mean classification accuracy and the lowest difference between classification accuracies per class. Possible windows were extracted from the motor imagery window by considering all trials of phase 1.

Finally, at the end of the experiments, all data were processed offline to classify all data and assess the related accuracy. Differently from above, an artifact removal technique was introduced as a pre-processing step preceding the processing pipeline discussed above. This consisted of the artifact subspace reconstruction (ASR) technique, which was applied to raw signals during offline processing [38]. This is a relatively recent technique for artifact removal exploited here to prepare data prior to feature extraction and classification. ASR uses an artifact-free data segment as a baseline and then corrects the original data by calculating a covariance matrix and retrieving statistics to identify and remove artifacts. Notably, its usefulness for an eight EEG channels setup is supported by previous studies [39].

The ASR was applied by means of EEGLAB, a MATLAB© open-source toolbox for EEG analysis developed by Delorme and Makeig in 2004 [40]. Notably, the plug-in for cleaning raw data was specifically used.

### 2.4. Outcome Measures

To evaluate the usability of the proposed system and the participants’ imaginative abilities, the following questionnaires were administered to participants of both groups:

- MIQ-3 [41]: this is the most recent version of the movement imagery questionnaire [42] and of the revised movement imagery questionnaire [43]. It is a 12-item questionnaire to assess an individual’s ability to imagine 4 movements using internal visual imagery, external visual imagery, and kinaesthetic imagery. The rating scales range from 1 (very difficult to see/feel) to 7 (very easy to see/feel). The MIQ-3 has good psychometric properties, internal reliability, and predictive validity.
- SUS (system usability scale) [44]: this is one of the most robust and tested psychometric tools for user-perceived usability. The SUS score consists of a value between 0 and 100, with high values indicating better usability. According to Bargor et al. [45], it is possible to adopt a 7-point adjectival scale (from “worst imaginable” to “best imaginable”) for the SUS score. Another variation, proposed in [46], is to consider the score in terms of “acceptable” (value above 70) or ”not acceptable” (value below 50). The range from 50 to 70 is instead “marginally acceptable”.
- NASA-TLX (acronym for NASA task load index) [47]: it is a subjective, multidimensional evaluation tool that assesses the perceived workload while performing a task or an activity. The original version also includes a weighting scheme to account for individual differences. However, the most common change made to the questionnaire is the elimination of these weights in order to simplify its application [48]. In this work, it was administered without weights.
- UEQ-S (user experience questionnaire—short form) [49]: a standardized questionnaire to measure the user experience of interactive products. It distinguishes between pragmatic and hedonic quality aspects. The first describes interaction qualities that relate to tasks or goals the user wants to achieve when using the product. The second describes aspects related to pleasure or enjoyment while using the product. Values between −0.8 and +0.8 represent a neutral evaluation of the corresponding scale, values greater than +0.8 represent a positive evaluation, and values lower than −0.8 represent a negative evaluation.

The MIQ-3 was administered twice: before the first experimental session and at the end of the last experimental session. In contrast, the other questionnaires were administered only at the end of the experimental sessions. In addition, during each experimental session, the participants were also given a short interview to assess their physical and mental state. This interview was adapted from the questionnaire proposed in [50], with some modifications needed to introduce aspects associated with neurofeedback [16].

### 2.5. Statistical Analysis

To compare classification accuracies between sessions and groups, a repeated-measures ANOVA test was used under the assumption of normally distributed data and homoscedasticity. The Jarque–Bera test was exploited to check for the normality assumption. The homoscedasticity was tested by means of the Bartlett’s test. In case of a violation for the assumption of homoscedasticity, it was possible to apply a Welch’s correction before applying the ANOVA. Meanwhile, the Kruskal–Wallis non-parametric test was used instead of the ANOVA when data were not normally distributed.

The comparison of MIQ-3 scores between the two groups and the two endpoints (before starting and at the end of the sessions) was conducted via the Mann–Whitney U test [51]. In addition, a Wilcoxon signed-rank test was used to compare paired data of the MIQ-3 scale within each group (control and neurofeedback). Similarly, a comparison between the two groups was carried on in terms of SUS, NASA-TLX, and UEQ-S scores at the end of the sessions. In each case, test-specific assumptions were checked before applying the test.

The statistical analyses were performed using MATLAB (version 2021b), and the significance level for them was set by α = 5% (the probability of a false negative or type-I error).

## 3. Results

Results are reported in this section after commenting on the sample of participants to the experimental campaign. Experimental data were analyzed in accordance with the methods of Section 2. Then, classification accuracies were exploited to assess the performance of the system and to describe its limits. Neurophysiological changes were also evaluated for each subject. The results are discussed in conjunction with answers to the questionnaires, especially to address the usability of the system in tele-rehabilitation. Additional details regarding the results are reported in the Supplementary Material section at the end.

### 3.1. Participants

A sample of 27 healthy volunteers was enrolled in the study (mean age: 26, standard deviation: 2). The study was approved by the Ethical Committee of Psychological Research of the Department of Humanities of the University of Naples Federico II, and all the participants provided a written informed consent before starting the experiments.

To investigate multimodal feedback, roughly half of the participants were assigned to the “control group” and half to the “neurofeedback group”. The two groups were balanced by age. In the control group, four subjects were males and nine were females. In the neurofeedback group, eight subjects were males and six were females. All participants used the wearable system with dry sensors while seated in front of a display for visual indications and eventual feedback. Participants with affected motor and/or cognitive functions were excluded. However, it is worth mentioning that a subject (C08) reported past epileptic seizures during childhood.

Most subjects were right-handed with the exception of two left-handed subjects per each group and one ambidextrous subject in the neurofeedback group. More than 60% of participants in the neurofeedback group practiced sport, while the participants in the control group practicing sport were less than 40%. No participant played sport at a professional level. More than 50% of participants already had experienced some BCI paradigms, and some subjects also had previous experience with neurofeedback. Such information is detailed in Table 1 along with a summary of previous information about sex, handedness, and practicing sport.

### 3.2. System Performance

**Figure 5:**
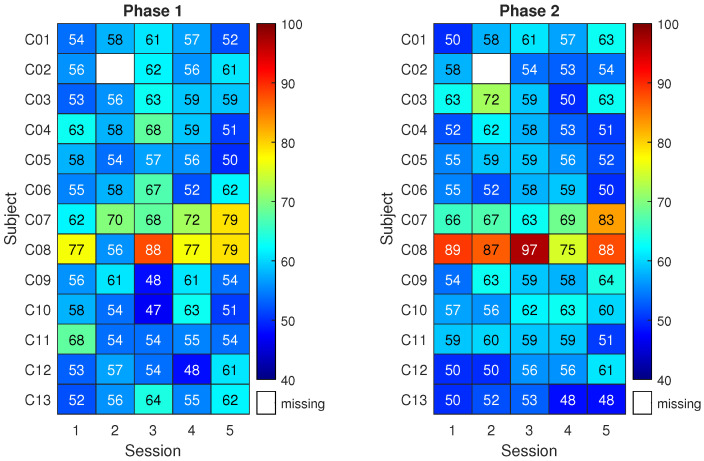
Control group: mean classification accuracy using the best 2-second window.

Classification results for the control group are shown in Figure 5. The matrix on the left reports the classification accuracy obtained on the first three runs of pure motor imagery (phase 1) across five sessions (*x*-axis) and for the thirteen subjects (*y*-axis). The matrix on the right reports the analogous results for the last three runs of pure motor imagery (phase 2). Higher classification accuracy is indicated by a red color. Meanwhile, a white space refers to a missing result caused by corrupted data or a skipped session.

Given that 90 trials were used for each classification result, the classification accuracy of a random classifier would be modeled by a binomial distribution with a mean equal to 50% (the well-known chance level) and a 95% coverage interval spanning from 40% to 59% (related to the number of trials) [52]. Notably, this implies that only classification accuracy values above 59% can be considered non-random with an α = 5%. Therefore, the classification accuracy for subjects in the control group was compatible with randomness except in a few cases. Overall, the highest mean classification accuracy across subjects was about 62% with 3% associated type A uncertainty and it was obtained in phase 2 of session 2 and phase 1 of session 3.

Only subjects C07 and C08 do not belong to the general trend. Notably, the classification accuracies exceed 70% in several cases, an empirical threshold for acceptable performance in motor imagery. Interestingly, C08 was the participant reporting past epileptic seizures.

**Figure 6:**
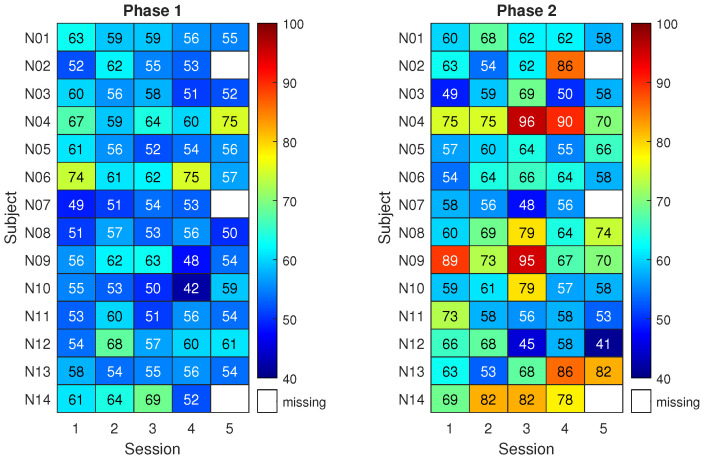
Neurofeedback group: mean classification accuracy using the best 2-second window.

Figure 6 shows the classification results for the neurofeedback group. The matrix on the right refers to three runs with neurofeedback (phase 2 for the neurofeedback group).

The results of phase 1 for the neurofeedback group appear similar to those of the control group, with classification accuracies close to the chance level. Nonetheless, 8 subjects out of 14 exceeded the 70% accuracy threshold at least once during phase 2. In more detail, by individually considering the sessions, the average improvement in classification accuracy due to neurofeedback ranges from 5% to 12%. The subjects reached the respective peak accuracies in different sessions. This led to a maximum average classification accuracy among subjects of 69% with 3% uncertainty.

Statistical testing suggested that the highest classification performance of the neurofeedback group in phase 2 did not differ significantly from the highest of the control group, though it was 7% higher on average. Instead, a statistically significant difference between the two groups was found when focusing on the third session of phase 2 (*p* < 0.05). Moreover, classification accuracy in phase 2 was significantly higher than that of phase 1 in the fourth session of the neurofeedback group (*p* < 0.005). Finally, when comparing all the classification accuracies (all subjects and all sessions) of the neurofeedback group with those of the control group, the improvement given by neurofeedback in phase 2 is statistically significant (*p* < 0.005).

### 3.3. Questionnaires

As mentioned in Section 2, the MIQ-3 was administered twice to each subject, i.e., before the first experimental session and at the end of the experimental sessions. On a scale from 1 to 7, the mean scores were above 5 already at the first endpoint, with only one exception, kinesthetic imagery, whose mean score equaled 4 for both groups. This implies that subjects generally considered it easy, or at least not difficult, to see/feel the involved movements. The Wilcoxon signed-rank test did not produce statistically significant variations in the MIQ-3 paired scores within each group. The same applies to the Mann–Whitney U test when considering differences between the two groups before and after the experiments.

The SUS scores suggest that the system was considered acceptable by both groups (above 70). Specifically, the results are equal to 78 ± 10 and 75 ± 11 for the control and neurofeedback groups, respectively. In addition, the overall results of the UEQ-s equaled 1.60 ± 0.64 for the control group and 1.70 ± 0.80 for the neurofeedback group. No statistically significant differences between the groups were detected (*p* = 0.40 for SUS and *p* = 0.98 for UEQ-s).

**Figure 7:**
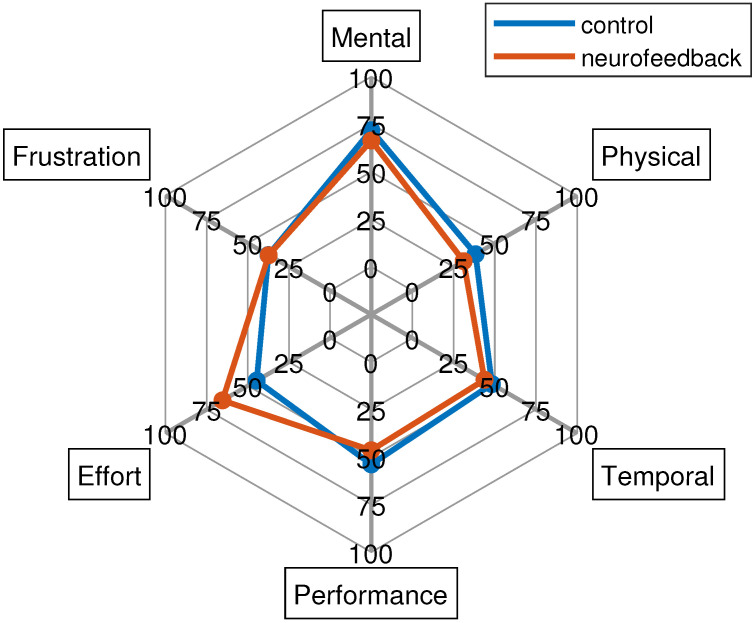
NASA-TLX results for both control and neurofeedback groups.

Finally, the NASA-TLX results are reported in Figure 7. This shows similar subscale results for both groups with the exception of the effort. In particular, the Mann–Whitney U test found statistically significant differences for the latter dimension between the two groups (*p* < 0.05) indicating that the neurofeedback group perceived that there was more effort required than the control group, which was anticipated due to the need to engage with neurofeedback. The mental demand was high (around 75 for both groups), while the frustration level, performance, and temporal and physical demand were low.

## 4. Discussion

In this concluding section, the results in terms of the system performance and its acceptability by healthy users are thoughtfully discussed. Next, how the present work discloses the possibility of tele-rehabilitation is commented on by relying on the current results to address future steps. Overall, both the limitations and strengths of the proposed system are considered in aiming to target the rehabilitation field.

### 4.1. System Features and Acceptability

Motor-imagery-based BCIs present the possibility of novel rehabilitation paradigms, either substituting or supplementing current therapy protocols. This technology can be an option for safe and economically viable home-based therapies.

However, several training sessions are typically required to successfully control such a BCI and, as a well-known problem in the literature, BCI illiteracy specifically prevents its widespread adoption. In such a framework, this study investigated the usage of neurofeedback to help a user to successfully control the system in few sessions while stressing daily-life and tele-rehabilitation requirements. As key aspects, the foreseen applications led to the adoption of a wearable and portable EEG with dry sensors, a wearable and portable actuator for the haptic feedback, and an easy-to-use software application including the visual feedback.

Indeed, using the dry sensors increased the comfort for the participants mostly by avoiding the usage of conductive gels. However, the signal-to-noise ratio of the EEG data was generally lower than the one associated with wet sensors. This appeared especially true if the user had medium to long hair. For instance, EEG signals were more affected by artifacts when using dry sensors. The main artifacts superimposed on the EEG signal were heartbeats (especially at O1 and O2), breathing, ocular artifacts, and sweat artifacts (especially at F1 and F2). Furthermore, unlike wet sensors [16], vibration-induced artifacts occasionally appeared when the feedback was delivered by the haptic suit. Although ASR applied offline removed most artifacts, the suit vibration had to be kept under control during online experiments, mostly by limiting its vibration intensity. This suggests that a different type of haptic feedback should be explored in the future.

In the proposed system, feedback was implemented in a non-immersive extended reality by simultaneously providing multiple sensory stimulation, namely the haptic and visual modalities. With respect to unimodal feedback, a greater impact on classification performance was expected [16]. Moreover, the multisensory stimulation aimed to increase users’ engagement. The resulting mean improvements (on the subjects) are in accordance with the previous evidence, which suggested that such feedback would have led to an about 6% to 8% improvement in classification accuracy if compared to the absence of feedback. In particular, a 7% increase was highlighted between the control group and the neurofeedback group, while the mean improvement between the two phases for the neurofeedback group ranged from 5% to 12%. Therefore, although only eight dry sensors were employed, the use of multimodal feedback led to an increase in system performance. In comparison, the subjects of the control group showed no significant improvement across the sessions, with the only exceptions of subjects C07 and C08, who achieved good results even without any feedback.

**Figure 8:**
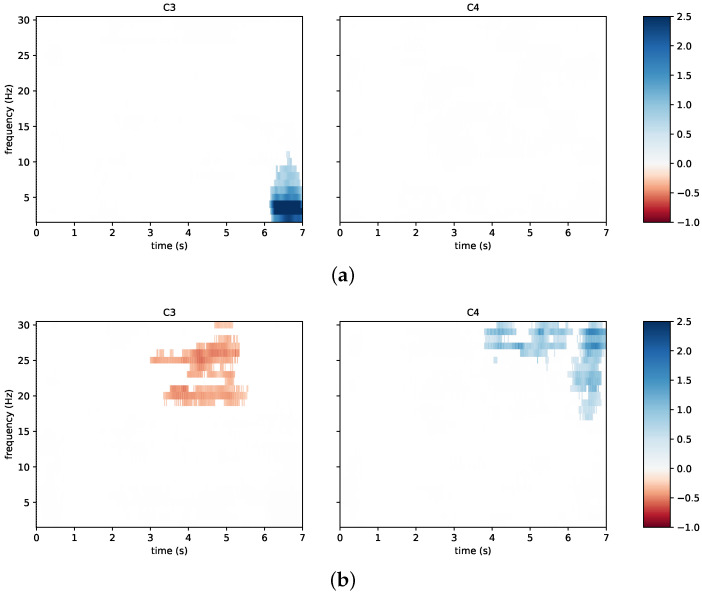
Time/frequency maps for a poorly performing subject from the neurofeedback group: (**a**) left hand imagery; (**b**) right hand imagery. The channels C3 and C4 are taken into account. Event-related desynchronization is depicted in red and event-related synchronization in blue.

The results in terms of classification accuracy can also be supplemented with physiological information for neurophysiological changes. Notably, in accordance with the discussed literature, event-related spectral perturbation was investigated. To this aim, Figure 8 reports time/frequency maps for the first session of subject N09 from the neurofeedback group. The figure focuses on the channels C3 and C4 in the cases of left hand imagery (Figure 8a) and right hand imagery (Figure 8b). The subject reached a low classification accuracy in this first session and, at the same time, a desynchronization only appears in the beta band for the right hand motor imagery on C3, while the same phenomenon does not appear for the left hand imagery.

**Figure 9:**
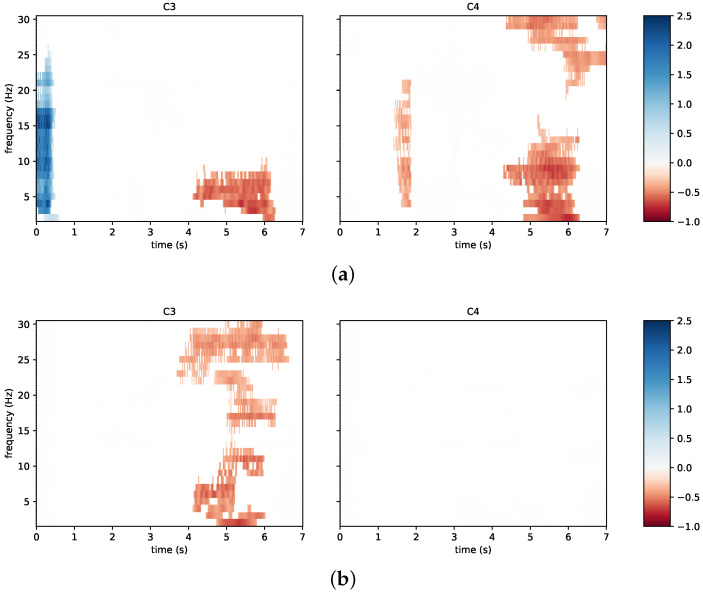
Time/frequency maps associated with the best accuracy result of the same subject from Figure 8: (**a**) left hand imagery; (**b**) right hand imagery. The channels C3 and C4 are taken into account. Event-related desynchronization is depicted in red and event-related synchronization in blue.

Figure 9 reports the time/frequency maps obtained in a different experimental session, in which the same subject reached the highest classification accuracy during neurofeedback (third session of N09). In such a case, and in accordance with the literature [53,54], left hand motor imagery is associated with a bilateral desynchronization, (Figure 9a) while right hand motor imagery is associated with a contralateral desynchronization (Figure 9b). Moreover, the timing of the event-related spectral perturbation is compatible with the best 2.00 s wide window selected in calculating the classification accuracy. Notably, the best window for this subject in the third session was from 4.00 s to 6.00 s.

**Figure 10:**
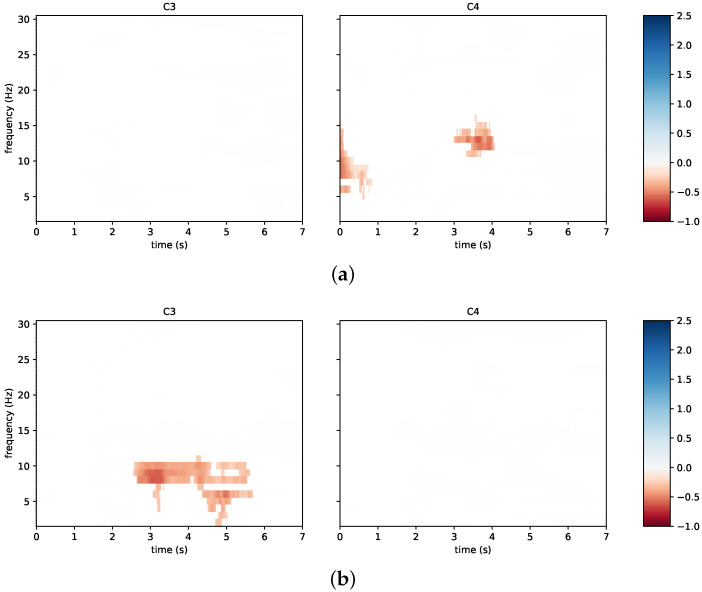
Time/frequency maps associated with a subject of the control group reaching high classification accuracy: (**a**) left hand imagery; (**b**) right hand imagery. The channels C3 and C4 are taken into account. Event-related desynchronization is depicted in red and event-related synchronization in blue.

The time/frequency maps representative of the neurofeedback group were also compared with those of the subject C07 from the control group. In particular, this subject was taken into account because it reached one of the highest classification accuracies. For instance, with respect to the last experimental session, a contralateral desynchronization in the 10 Hz to 15 Hz band appears for left hand motor imagery (Figure 10a) and a contralateral desynchronization also appears for right motor imagery (Figure 10b). Notably, the best 2.00 s wide window for this subject and for this session was 2.75 s to 4.75 s, where both neurophysiological phenomena occur.

With the short interview administered during each experimental session, it was also possible to monitor the subjects’ mental and physical state during the sessions, as well as the type of imagined movement. In general, the most common imagined movements were squeezing a ball, moving the arm, tapping, grasping an object, dribbling, or playing piano. Nonetheless, it is worth noting that 6 out of 13 subjects in the control group changed the type of movement imagined during the sessions and, among these, three subjects also switched between internal, external, and kinaesthetic imagery. Seven out of fourteen subjects in the neurofeedback group changed the type of imagined movement during the sessions and, among these, four subjects changed between internal, external, and kinaesthetic imagery. According to the results, one can suspect that low-performance levels would also be caused by changes in the imagined movement during the sessions, especially when feedback was not provided. Therefore, such an aspect should be more rigorously kept under control in future protocols.

Overall, the SUS and UEQ-s questionnaires showed that the system is user-friendly, and subjects of both groups had a positive experience. This was not obvious with dry sensors because these require proper pressure to obtain a suitable electrode–skin contact. In turn, this could have implied pain and affected the overall system, whereas motor imagery requires users’ deep concentration on the task. Contrary to expectations, the MIQ-3 did not show differences between groups and sessions as the imagination scores reported by the participants were high both before and after the experiments. A possible explanation would be that such a questionnaire is not directly linked to left/right hand movements, which are common motor imagery tasks. Therefore, its scale may not be sensitive enough for the tasks of this work, although no other standard scale exists for this purpose. Finally, the NASA-TLX effort was statistically higher for the neurofeedback group. This result may be explained by the constant demand required by these subjects, who received a response to their mental state during the online experiment.

### 4.2. Toward Tele-Rehabilitation

Several studies demonstrate the benefits of motor-imagery-based systems for patients with variegated neurological diseases [55,56,57]. In these cases, neurophysiological signatures of motor imagery may undergo changes following brain trauma [58]. Indeed, such patients may present various medical conditions posing challenges for BCI-based tele-rehabilitation. These include cognitive impairment and different sensory deficits [59]. Moreover, it is crucial to recognize that brain reorganization takes place after lesions in the central nervous system. This can significantly impact the recovery of lost sensory and motor functions [55]. Therefore, the integration of motor imagery with neurofeedback assumes significance as an essential component of rehabilitation procedures. Another essential element that should be considered in BCI-based tele-rehabilitation is the wide spectrum of needs of patients in terms of usability and applicability. Indeed, factors such as frustration, cognitive load, and fatigue can significantly impact the patient’s experience and their interaction with the system.

Despite its exploratory nature, this work offers valuable insights into BCI-based tele-rehabilitation. Firstly, the proposed system allows for home use thanks to its features, e.g., the employment of dry electrodes. Using the system at home also discloses the possibility to reduce the duration of rehabilitation sessions while increasing their number. In addition, our results in mental fatigue could be useful to direct future therapy applications, especially for patients with cognitive impairments. Finally, the present study suggested that animated objects or better limbs could aid in imaging movements. This aspect is essential for patients with motor disabilities, which may have more difficulties in maintaining vivid motor images with respect to healthy subjects [60,61]. The addressed improvements will be possible thanks to the wearability and the rehabilitation benefits of the proposed motor-imagery-based BCI. Overall, the investigated system will address tele-rehabilitation purposes because of the perceived usability and the substantial improvement in classification accuracy revealed in the neurofeedback group with respect to the control group.

A limitation of this study in tele-rehabilitation applications is that the multimodal proposed feedback was positively biased. Nonetheless, this can be enhanced with an adaptive bias to optimize system performance and patient learning [62], and future development could also focus on improving the classification algorithm to enhance performance across sessions and deliver better feedback [63]. Although multiple sessions were carried out already with healthy subjects, it is worth emphasizing patients would require even more training sessions to gain proper control over the BCI system and obtain benefits from therapy [64].

## Figures and Tables

**Table 1:** Summary of participants’ information for control and neurofeedback groups. BCI experience: experience with brain–computer interfaces in active paradigms, passive paradigms, reactive paradigms, or multiple paradigms or no experience. NF experience: previous experience with neurofeedback or no experience.

	Control	Neurofeedback
Sex	male: 31%, female: 69%	male: 57%, female: 42%
Handedness	right: 85%, left: 15%, both: 0%	right: 79%, left: 14%, both: 7%
Practicing sport	yes: 38%, no: 62%, professional: 0%	yes: 64%, no: 36%, professional: 0%
BCI experience	no: 38.5%, active: 8%, passive: 15%,	no: 43%, active: 7%, passive: 21%,
	reactive: 0%, multiple: 38.5%	reactive: 0%, multiple: 29%
NF experience	yes: 46%, no: 54%	yes: 36%, no: 64%

## Data Availability

The dataset is publicly available at https://metroxraine.org/contest-dataset (accessed on 12 Febraury 2023).

## Supplementary Materials

The results presented here can be reproduced by exploiting the code published at https://github.com/anthonyesp/neurofeedback (accessed on 12 Febraury 2023).
